# Implementation of contingency management with women engaging in polysubstance use

**DOI:** 10.1186/s13722-025-00590-x

**Published:** 2025-08-08

**Authors:** Kathleen M. Ward, Adam W. Carrico, Daniel Vader, Reneé H. Moore, K. Rivet Amico, Allison K. Groves, Scarlett L. Bellamy, Susan G. Sherman, Douglas Krakower, Silvana Mazzella, Alexis M. Roth

**Affiliations:** 1 Dornsife School of Public Health, Department of Community Health and Prevention, Drexel University, Philadelphia, PA USA; 2 Robert Stempel College of Public Health and Social Work, Department of Health Promotion and Disease Prevention, Florida International University, Miami, FL USA; 3 Dornsife School of Public Health, Department of Biostatistics, Drexel University, Philadelphia, PA USA; 4 Department of Health Behavior and Health Education, University of Michigan School of Public Health, Ann Arbor, MI USA; 5 Department of Biostatistics, Boston University School of Public Health, Boston, MA USA; 6 Department of Health, Behavior and Society, Johns Hopkins Bloomberg School of Public Health, Baltimore, MD USA; 7 Department of Population Medicine, Harvard Medical School, Boston, MA USA; 8 Division of Infectious Diseases, Beth Israel Deaconess Medical Center, Boston, MA USA; 9 Prevention Point Philadelphia, Philadelphia, PA USA

**Keywords:** Contingency management, Women who use drugs, Polysubstance use, Opioid, Stimulant

## Abstract

**Background:**

Contingency management (CM) is an effective intervention that provides financial incentives as positive reinforcement for reducing opioid or stimulant use. However, it has not been tested in populations of women who inject drugs (WWID) engaging in polysubstance use.

**Methods:**

We aimed to compare the feasibility of two CM protocols designed to encourage illicit stimulant and opioid abstinence among WWID participating in an ongoing HIV prevention trial. Participants completed a 3-month CM period during which they submitted thrice weekly urine toxicology screenings (UTOX). In the ‘abstinence from stimulants and opioids’ protocol, participants received a $5 USD incentive when metabolites of stimulants and opioids were not detected in urine. In the ‘partial-abstinence protocol’, they received a $5 USD incentive when metabolites of stimulants or opioids were not detected, thus doubling the potential incentive obtained each visit. Women also received scaling bonuses after three consecutive negative UTOX ($5-$15 USD). We used descriptive statistics to summarize the total number of (1) UTOXs completed and (2) bonuses distributed. Rates of engagement per person per month were calculated (i.e., total number of completed UTOX/3 months*24 participants). Rates of engagement were compared by CM protocol period.

**Results:**

Participants were primarily White women (67%) with an average age of 47 years. Self-reported polysubstance use was common (96%) with women reporting injecting an average of 5 times daily (Interquartile Range: 2–7). Participants (*N* = 24) collectively submitted 177 UTOX during their 3-month CM periods. Rates of non-reactive UTOX results were slightly higher in the partial-abstinence protocol compared to the abstinence from stimulants and opioids protocol (2.9 per month versus 1.0 per month). More bonuses were earned in the partial-abstinence protocol (0.50 bonuses per participant per month) compared to the abstinence from stimulants and opioids protocol (none). There were no study related adverse events in either protocol group during the CM period.

**Conclusions:**

Findings demonstrate the feasibility of a CM protocol that provided financial incentives for partial abstinence, periods with documented stimulant or opioid abstinence, as well as abstinence to both, without the occurrence of iatrogenic effects. Future research focusing on CM protocols with more flexible incentive structures remains critical.

**Trial registration:**

NCT05192434.

## BACKGROUND

The “fourth wave” of the overdose crisis in the United States is characterized by polysubstance use, especially the use of illicitly manufactured synthetic opioids (e.g., fentanyl) and stimulants (e.g., methamphetamine) concurrently or within a short period of time [1, 2]. Between 2012 and 2022, nearly two-thirds of overdose deaths were among people engaging in polysubstance use [3]. Women were overrepresented in these deaths compared to men, despite having lower rates of drug use overall [3, 4]. Scalable interventions that target opioid and stimulant use disorders simultaneously are urgently needed to prevent fatalities, and address gender-based overdose disparities.

Although there are safe and effective medications to treat opioid use disorder [5], behavioral interventions are currently considered first-line treatments for stimulant use disorders [6–8]. Among them is contingency management (CM), an evidence-based intervention [9, 10] that provides financial incentives such as cash, vouchers, or lottery drawings as positive reinforcement for performing target behavior(s), such as drug abstinence [11]. Rewards typically escalate the longer the participant sustains the target behavior [11], and many protocols reset when a participant relapses or misses an appointment [12, 13].

CM protocols with punitive reset components or protocols that require abstinence from several substances simultaneously may not be realistic for many patients [14, 15]. In a prior meta-analysis, CM interventions targeting abstinence from multiple substances yielded small to moderate effects (Cohen’s d = 0.42) [9]. While CM for opioid use among people in methadone treatment (Cohen’s d = 0.65) or stimulant use (Cohen’s d = 0.66) achieved more robust effect sizes, indicating moderate reductions in use. Thus, a growing body of research suggests CM models that incentivize each target behavior separately may be needed to address the polysubstance use that is associated with greater overdose deaths among women [16].

This study evaluates two CM protocols designed to reduce polysubstance use among a non-treatment seeking sample of women who inject drugs (WWID). The first protocol required non-reactive urine specimen for opioids *and* stimulants to receive incentives while the second utilized a more flexible approach that rewarded either a stimulant or opioid non-reactive sample (or both), without the participant being required to select one or the other at baseline. The second protocol was developed in response to low engagement with the first protocol. Here, we compare participant engagement (i.e., completion of the urine toxicology visits) in each protocol to provide some indication of their safety and feasibility.

## Methods

### Eligibility

Participants represent a subset of women enrolled in *TIARAS* between June 2022-June 2023 (*n* = 40). *TIARAS* is an ongoing randomized controlled trial testing whether a trauma-informed writing intervention can boost and extend the impact of CM on HIV acquisition risk among WWID in Philadelphia (PA, USA) [17]. *TIARAS* participants are English-speaking, cisgender women, age 18 or older, who reported using injection drugs within 6-months and received a prescription for pre-exposure prophylaxis (PrEP) within 30-days (confirmed via medical records).

After enrollment, *TIARAS* participants immediately begin a 3-month CM period that includes thrice weekly urine toxicology screenings (UTOX) assessing metabolites of opioids and stimulants using ACCURATE™ 10 Panel Rapid Drug Test Cup, CLIA Waived. As part of the *TIARAS* protocol, participants are required to provide at least two urine specimens during the first 6-weeks (run-in period) before being randomly assigned to complete either four neutral or trauma-focused essays within the CM period. Each essay is compensated at $20 USD.

*TIARAS* participants were included in this analysis if they completed the 3-month CM period and provided at least three urine specimens (two during the run-in and at least one post-randomization). For this reason, nine participants were dropped from the analytic sample. Of the remaining 31 women, 6 were exposed to the abstinence from stimulants and opioids protocol, 18 were exposed to the partial abstinence protocol, and 7 were exposed to both. For ease of comparison, the analytic sample includes participants only exposed to the abstinence from stimulants and opioids *or* partial abstinence protocol (*N* = 24).

### CM protocol

The CM protocol in *TIARAS* was guided by an implementation science, stepped-wedge trial testing a CM intervention with female entertainment and sex workers who use stimulants. In this trial, we observed reductions in stimulant use after CM with a cognitive-behavioral aftercare group intervention [18, 19]. In *TIARAS*, the initial abstinence from stimulants and opioids protocol required participants to refrain from using both stimulants and opioids to receive incentives. Participants received a $5 USD incentive for each urine sample that was non-reactive for stimulants *and* opioids, with the potential to receive escalating bonuses for 3 consecutive non-reactive UTOX (weeks 1–4 $5 USD; weeks 5–8 $10 USD; weeks 9–12 $15 USD). There were no penalties (e.g., re-setting incentive scales) for reactive UTOX or missing an appointment (total possible incentive earnings: $240). Engagement with the initial protocol was low, with only 9% of CM UTOX visits completed. In response, after 5 months of data collection (June to October 2022), we modified the CM incentive structure to provide women more opportunities to earn incentives.

In the modified “partial abstinence” CM protocol, participants received a $5 USD incentive for urine specimen that were non-reactive for opioids *or* stimulants and were eligible to receive both incentives during a single visit (i.e., a non-reactive UTOX for both opioids and stimulants earned $10 USD). Participants had access to the same escalating bonuses structure as before but could earn double the bonuses for non-reactive UTOX for both opioids and stimulants (weeks 1–4 $5–10 USD; weeks 5–8 $10–20 USD; weeks 9–12 $15–30 USD). This change effectively doubled the total possible CM incentive pool (from $240 to $600).

In addition to incentives for drug abstinence, participants could also be compensated for adherence to pre-exposure prophylaxis though the intervention, which will be described elsewhere when *TIARAS* is complete.

### Staff training and fidelity monitoring

All study staff receive extensive training on performing study procedures, which are detailed in a standard operating procedure manual. Training components relevant to the contingency management protocol include: (1) maintaining privacy and confidentiality including HIPAA, human subjects protections, and data safety protections; (2) obtaining informed consent and ensuring participant understanding of the CM payment schedule; (3) properly handling, testing, and disposing of biological specimens; (4) sharing urine toxicology screening results with participants in a non-stigmatizing way; (5) consistent data entry at the time of study visit.

All staff shadow and perform mock study visits prior to beginning data collection. More experienced staff members observe and provide support during initial data collection visits. Staff also engage in ongoing group and individual supervision meetings where quality assurance, problem-solving, fidelity monitoring, cultural sensitivity, and harm reduction are discussed.

### Variables and measures

**Sociodemographic information and substance use characteristics** were collected in the baseline survey (compensation=$30 USD) and included current living situation (stably housed v. unhoused [shelter, car, street, abandoned building, or facility]). Annual income (continuous) was a composite of various income sources: full/part-time work, social security payments, public assistance, and odd jobs/hustling (i.e., selling sex and shoplifting).

**Substance use in the past 3 months** was assessed at baseline using The Alcohol, Smoking and Substance Involvement Screening Test and categorized as any stimulant use (cocaine, methamphetamines, or prescription); any opioid use (street opioids [fentanyl, heroin], or non-medical use prescription opioids); or polysubstance use (both stimulant and opioid use). We also collected self-report information regarding medication to treat opioid use disorder in the last 6 months (y/n); recency of injection drug use (daily, weekly, or over one month ago); average daily number of injections; and lifetime overdose history (ever v. never).

Biological markers included the UTOX results during the 3-month CM period. A non-reactive UTOX for stimulants was defined as a urine sample in which metabolites of cocaine and methamphetamine were not detected. A non-reactive UTOX for opioids was defined as a urine sample in which metabolites of opiates and fentanyl were not detected.

### Analyses

We used descriptive statistics to summarize participant sociodemographics, medication to treat opioid use disorder, and substance use. Frequencies are reported for: (1) the total number of UTOX completed across the sample; (2) the number of non-reactive UTOX for stimulants across the sample; (3) the number of non-reactive UTOX for opioids across the sample; (4) the number of non-reactive UTOX for stimulants *or* opioid negative across the sample; and (5) the number of bonuses distributed. Rates of engagement per person per month were calculated (i.e., total number of completed UTOX/3 months*24 participants). Rates of engagement were then compared by CM protocol period.

### Ethical considerations

The Drexel University and City of Philadelphia Department of Public Health institutional review boards approved study procedures.

## Results

### Participant sociodemographics

Participants (*N* = 24) collectively submitted 177 UTOX during their 3-month CM periods. As shown in Table 1, most women were non-Hispanic White, unstably housed, and with annual incomes below the federal poverty limit for a single person. Self-reported polysubstance use was common (*n* = 23, 96%) with most women reporting injecting daily (median number of injections per day: 5 [Interquartile Range: 2–7]). Three-fourths (*n* = 18, 75%) of women experienced at least one unintentional drug overdose in their lifetime. Urine toxicology screening visits lasted approximately 15 min during which urine is processed, and results are shared with participants. Any adverse events are also reported during this time.

**Table 1:** Participant sociodemographics

	Total (*N* = 24)	Abstinence from stimulants and opioids protocol (*n* = 6)	Partial abstinence protocol (*n* = 18)
**Median age** (IQR)	47 (37–2.5)	41 (32–53)	47 (39–52)
**Race**			
American Indian/Alaska Native	1 (4.2%)	0 (0%)	1 (5.6%)
Black or African American	3 (12.5%)	1 (16.7%)	2 (11.1%)
White	16 (66.7%)	4 (66.7%)	12 (66.7%)
Not reported	1 (4.2%)	0 (0%)	1 (5.6%)
More than one race^1^	3 (12.5%)	1 (16.7%)	2 (11.1%)
**Ethnicity**			
Hispanic	3 (12.5%)	1 (16.7%)	2 (11.1%)
Non-Hispanic	21 (87.5%)	5 (83.3%)	16 (88.9%)
**Educational attainment**			
Less than high school	8 (33.3%)	1 (16.7%)	7 (38.9%)
High school graduate/GED	9 (37.5%)	4 (66.7%)	5 (27.8%)
Trade school or some college+	7 (29.2%)	1 (16.7%)	6 (33.3%)
**Annual income** (IQR)	$10,596 ($3,720-$17,634)	$11,364 ($2,400-$14,400)	$8,466 ($3,840-$17,892)
**Current living situation**			
Stably housed	8 (33.3%)	3 (50.0%)	5 (27.8%)
Unhoused	16 (66.6%)	3 (50.0%)	13 (72.2%)
**Taken medication for OUD (6 months)**	9 (37.5%)	3 (50.0%)	6 (33.3%)
**Self-reported drug use**^**2**^ **(3 months)**			
**Any stimulant use**	23 (95.8%)	5 (83.3%)	18 (100%)
Cocaine	20 (83.3%)	4 (66.7%)	16 (88.9%)
Prescription stimulants	4 (16.7%)	0 (0%)	4 (22.2%)
Methamphetamine	12 (50.0%)	2 (33.3%)	10 (55.6%)
**Any opioid use**	23 (95.8%)	5 (83.3%)	18 (100%)
Street opioids	22 (91.7%)	5 (83.3%)	17 (94.4%)
Prescription opioids	7 (29.2%)	0 (0%)	7 (38.9%)
**Polysubstance use**^**3**^	23 (95.8%)	5 (83.3%)	18 (100%)
**Recency of intravenous drug use**			
Not within month	3 (12.5%)	1 (16.7%)	2 (11.1%)
Weekly or less within month	3 (12.5%)	0 (0%)	3 (16.7%)
Daily within month	18 (75.0%)	5 (83.3%)	13 (72.2%)
**Average daily injections** (IQR)	5 (2–7)	6.5 (3–10)	4.5 (1–7)
**Lifetime overdose**	18 (75.0%)	5 (83.3%)	13 (72.2%)

^1^More than one race = American Indian/Alaska Native & White (*n* = 1); American Indian/Alaska Native & Black/African American & White (*n* = 2)

^2^The Alcohol, Smoking and Substance Involvement Screening Test (ASSIST)

^3^Polysubstance use is defined as both stimulant *and* opioid use in the past 3 months

### CM UTOX results

**Fig. 1:**
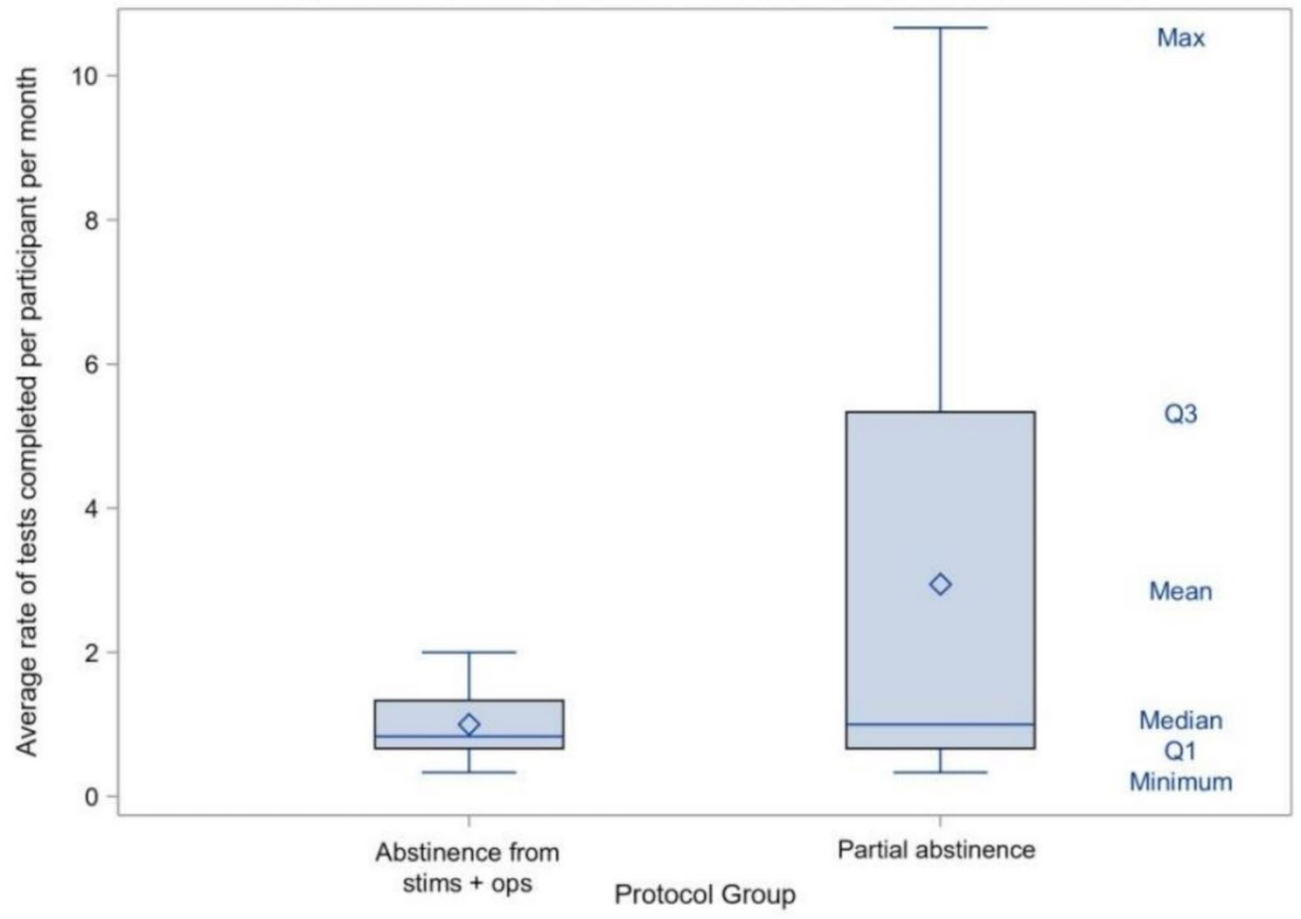
Rate of completed urine tests by protocol group

Across both CM protocols, approximately one-in-five UTOX appointments were completed (i.e., 177/821; 22%). The proportion of completed CM UTOX visits was nearly three-fold greater in the partial abstinence CM protocol (i.e., *n* = 159, 26%) versus the abstinence from stimulants and opioids CM protocol (*n* = 18, 9%). As shown in Fig. 1, the average rate of UTOX completed per participant was 2.9 per month in the partial abstinence CM protocol versus 1.0 per month in the abstinence from stimulants and opioids CM protocol.

The average rate of receiving a non-reactive UTOX result for stimulants and opioids per participant, per month was higher in the partial abstinence CM protocol compared to the abstinence from stimulants and opioids CM protocol (0.91 v. 0.11) (Table 2). The average rate of receiving either a non-reactive UTOX result for stimulants or opioids per participant, per month was higher in the partial abstinence protocol compared to the abstinence from stimulants and opioids CM protocol (2.13 v. 0.22). The average rate of receiving a non-reactive UTOX result for stimulants per participant, per month was higher in the partial abstinence CM protocol compared to the abstinence from stimulants and opioids CM protocol (1.65 v. 0.22). Finally, the average rate of receiving non-reactive UTOX result for opioids per participant, per month was higher in the partial abstinence CM protocol compared to the abstinence from stimulants and opioids CM protocol (1.39 v. 0.11). More bonuses were earned in the partial abstinence CM protocol (0.50 bonuses per participant per month) compared to the abstinence from stimulants and opioids CM protocol (none). There were no study related adverse events in either group during the CM period.

**Table 2:** Urine toxicology screening results from 12-week CM period by observation

Urine Screening Results by Observation	Total (*n* = 177)	Abstinence from stimulants and opioids protocol (*n* = 18)	Partial abstinence protocol (*n* = 159)
**No. tests stimulant and opioid non-reactive**	51 (28.8%)	2 (11.1%)	49 (30.8%)
Average rate non-reactive per participant per month	0.71	0.11	0.91
**No. tests stimulant or opioid non-reactive**	119 (67.2%)	4 (22.2%)	115 (72.3%)
Average rate non-reactive per participant per month	1.65	0.22	2.13
**No. of abstinence related bonuses received**	27 (15.3%)	0 (0%)	27 (17.0%)
Average rate of abstinence related bonuses per participant per month	0.38	0	0.50
**No. tests stimulant non-reactive**	93 (52.5%)	4 (22.2%)	89 (56.0%)
Average rate non-reactive per participant per month	1.29	0.22	1.65
**No. tests opioid non-reactive**	77 (43.5%)	2 (11.1%)	75 (47.2%)
Average rate non-reactive per participant per month	1.07	0.11	1.39

## Discussion

This study describes the implementation and preliminary impact of a CM intervention to address polysubstance use of opioids and stimulants among women who inject drugs outside of a treatment setting. Findings demonstrate the feasibility and safety of a CM protocol that provided financial incentives for partial abstinence - periods with documented stimulant *or* opioid abstinence, as well as abstinence to both. There was no evidence of iatrogenic effects of modifying the CM protocol to support partial abstinence. On the contrary, rates of non-reactive UTOX results and bonuses for week-long periods of abstinence were slightly higher in the modified partial abstinence CM protocol compared to the CM protocol that required abstinence from stimulants and opioids. Our findings are consistent with the contingency management literature in which more focused behavioral goals, such as targeting one drug versus multiple drugs, are more effective [9].

Our study responds to the urgent call by Khazanov and colleagues [16] to incorporate harm reduction frameworks into CM protocols, particularly in the context of today’s volatile drug market and the marked increase in polysubstance use [3]. Furthermore, harm reduction approaches supporting partial abstinence may have benefits beyond overdose prevention. For example, research using the National Survey on Drug Use and Health found that the prevalence of Hepatitis B, Hepatitis C, and sexually transmitted infections were more common among individuals reporting polysubstance use than those using only opioids [20]. This suggests that interventions to reduce stimulant use among individuals who also use opioids could have sexual health benefits. As health care providers continue to explore CM for SUD treatment [14], studies focusing on real-world implementation of responsive CM protocols with adequate incentive structures for women who use substances remain critical.

Importantly, the partial abstinence protocol not only had a different behavioral goal but also had a higher magnitude of incentive earnings. The potential earnings of $600 USD in the partial abstinence group is more comparable to other CM protocols, for example, the CM roll-out in California in which incentives totaled $599 USD [21]. The partial abstinence protocol incentives were double that of the abstinence from stimulants and opioids protocol. Therefore, it cannot be determined whether it was the higher incentive or the more flexible behavioral goals that increased CM engagement. However, results show a higher rate of urine screenings that are either stimulant or opioid non-reactive, compared to stimulant and opioid reactive indicating the behavioral goal may have been the main driver of increased engagement.

This research has several important limitations. The partial abstinence protocol was conceptualized after the abstinence from stimulants and opioids protocol failed to engage participants. Therefore, this analysis is comprised of a small, non-random, convenience sample that may not be generalizable to other populations. This limited sample also means we were underpowered to test for differences between protocol groups using inferential statistics. Yet, our descriptive findings provide strong preliminary evidence for the feasibility of a partial abstinence CM protocol. In addition, the way we operationalized the definition of polysubstance use means that we are only able to comment on when polysubstance use occurred, and not the timing (sequential or concurrent) which has important implications for overdose risk. Finally, it is unclear how UTOX results were impacted by drug market factors beyond the participant’s control. Adulterants are common in the Philadelphia drug supply so it’s possible that some positive results stem from unintentional consumption. We did not collect data on drug seeking behavior to unpack the frequency of this occurrence.

Future research could examine the partial abstinence protocol in mixed gender samples, since most of the gender-based CM literature is among men who have sex with men, and differences between genders have been largely understudied [22]. Men and women may have different experiences with CM, but the literature is mixed, and results are variable based on the drug and the structure of the CM protocol. For example, CM research related to nicotine cessation had a greater impact on women’s self-efficacy compared to men [23]. However, findings from alcohol use cessation programs finds no differences by sex [24]. More research is needed to understand if there are any differences in the efficacy in CM protocols by gender.

## Conclusions

Our findings provide strong preliminary support for the use of partial abstinence CM models. Women enrolled in the partial abstinence group provided more UTOXs and more occasions of week-long periods of abstinence, as evidenced by the number of bonuses distributed to this group compared to the abstinence from stimulants and opioids group. Moreover, we found that participation in the less restrictive protocol was not associated with iatrogenic effects, suggesting this more harm reduction-oriented approach is potentially more effective and equally safe. Future randomized controlled trials should test the comparative effectiveness and safety of different abstinence behavioral goals and CM protocols in larger samples that include treatment seeking individuals.

## Data Availability

The datasets generated and analyzed during the current study are not publicly available as enrollment for the parent study is still on-going. The full dataset will be available from the corresponding author on reasonable request at a later date.

## Abbreviations

CM: Contingency Management
WWID: Women Who Inject Drugs
PrEP: Pre-exposure Prophylaxis
UTOX: Urine Toxicology Screening
IQR: Interquartile Range
Q: Quartile
